# Postpartum Thoracic Endometriosis: A Case Report

**DOI:** 10.7759/cureus.83199

**Published:** 2025-04-29

**Authors:** Susan E Garber, Katherine S Lammers

**Affiliations:** 1 Obstetrics and Gynecology, Rochester General Hospital, Rochester, USA; 2 School of Medicine, St. George’s University, True Blue, GRD

**Keywords:** catamenial pneumothorax, endometriosis of the diaphragm, recurrent spontaneous pneumothorax, thoracic endometriosis, thoracic endometriosis syndrome

## Abstract

Thoracic endometriosis is a rare disease characterized by the presence of endometrial tissue within the thoracic cavity. Diagnostic findings, symptom presentations, and treatment methods have varied in defining thoracic endometriosis. Most criteria, however, require spontaneous pneumothorax within 72 hours of menses as the *sine qua non* for this diagnosis. This report describes a case of spontaneous pneumothorax from thoracic endometriosis in a postpartum female, a unique presentation not yet reported in the literature. The patient clinically improved with oxygen via a non-rebreather mask, and no additional intervention was required as the patient clinically improved on her own. Although there are many theories, there is no single theory that fully explains the pathogenesis of this disease. With further research on this disease, there is hope for a better understanding of the etiology and, subsequently, better treatment options.

## Introduction

Endometriosis is the presence of endometrial tissue, glands, and stroma outside the uterine cavity. The pelvis is the most common location of endometriosis, with a peak incidence in women aged 24-29 years [1]. The most common endometriosis location outside the abdominopelvic cavity is within the thoracic cavity [2]. Thoracic endometriosis is diagnosed when endometrial tissue is identified on histological examination from samples obtained via chest tube aspirates, thoracotomy, or bronchoscopy, revealing hormone receptor-positive endometrial stroma and glands. The term thoracic endometriosis syndrome is used when there are clinical manifestations of the disorder presenting with menstruation but not confirmed by histology [3]. The term catamenial is also used when symptoms present along with menses. Although there may be distinct definitions of the terms, they are all used interchangeably in the literature.

Thoracic endometriosis is a rare disease with an unknown incidence within the general population, with the peak incidence at the ages of 30-34 years. It is estimated that 50-84% of individuals with thoracic endometriosis also have pelvic endometriosis [2]. In a retrospective study of all patients at the University Hospital of Martinique with thoracic endometriosis syndrome studied from January 1, 2004, to December 31, 2020, the researchers determined the annual incidence of thoracic endometriosis syndrome was 1.1 cases per 100,000 with a prevalence of 1.2 per 1,000 females for an age range of 15-45 years [4]. The most common symptoms of thoracic endometriosis include pneumothorax, hemothorax, hemoptysis, and chest pain. Spontaneous pneumothorax within 72 hours of the onset of menses has been termed the *sine qua non* for a catamenial pneumothorax diagnosis [5]. This case describes an unusual presentation of thoracic endometriosis in a postpartum patient. Although the patient had a history of numerous spontaneous pneumothoraces before the current presentation, they all aligned with menses. However, the current spontaneous pneumothorax presented five days after the cesarean section delivery of her first child. The pathogenesis of thoracic endometriosis is poorly understood, emphasizing the importance of adding case reports to the literature. There are a variety of theories proposed, but none that fully explain the pathogenesis. It is possible that any hormonal change, not just menstruation, can spark endometriosis-related pneumothoraces.

## Case presentation

**Figure 1:**
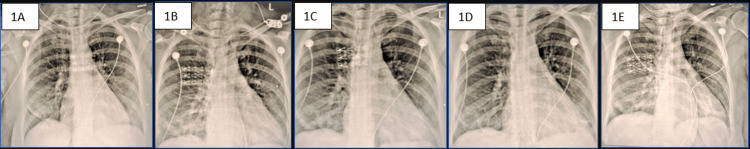
Serial chest X-rays. A: 6/19/24 at 4:09 am: mild pneumothorax on the right side. B: 6/19/24 at 7:58 am: stable-appearing right-sided pneumothorax, right lung atelectatic changes seen with right mild-to-lower lung zone, findings consistent with developing pneumomediastinum seen within the right paratracheal region. C: 6/19/24 at 15:01 pm: persistent right lower lung zone subpulmonic pneumothorax judged to be approximately 15%. Persistent right lower lung zone atelectatic change. (D) 6/19/24 20:22 pm: unchanged small right pneumothorax with underlying atelectasis. Sub-segmental atelectasis left lower lobe. E: 6/20/24 at 6:17 am: small right lower zone pneumothorax. No significant interval change.

A 40-year-old Puerto Rican female, with a past medical history of asthma (since 2013), seven previous spontaneous pneumothoraces associated with a popping sensation in her chest all aligned with menses (first episode 2015) status-post thoracotomy and pleurodesis on the right side (2015) and decortication (2016), myomectomy (2021), laparoscopic ovarian cyst removal, a negative workup of pelvic endometriosis (2021), and use of in vitro fertilization therapy for her pregnancy, presented to the emergency department five days after the cesarean section delivery of her first child, for which she was currently breastfeeding, with right-sided chest discomfort, difficulty breathing, and palpitations for one hour. The patient noted the discomfort felt similar to her past episodes of spontaneous pneumothoraces. On admission, the patient’s vitals were stable (temperature: 37.7°C, blood pressure: 163/67 mmHg, heart rate: 79 beats/minute, respiratory rate: 18 breaths/minute, and peripheral oxygen saturation: 99%). She had good air entry with decreased breath sounds in the right lower lobe, not prominent on the right flank and anterior chest. The patient’s initial chest X-ray showed a right lower lung pneumothorax, and electrocardiography was negative. Serial chest X-rays first showed a significant right lower lobe pneumothorax. A subsequent X-ray showed that it was larger initially with an apical pneumothorax. A subsequent X-ray showed suspected pneumomediastinum, which was not definitively apparent with right lower lobe atelectasis before resolving (Figure 1).

**Figure 2:**
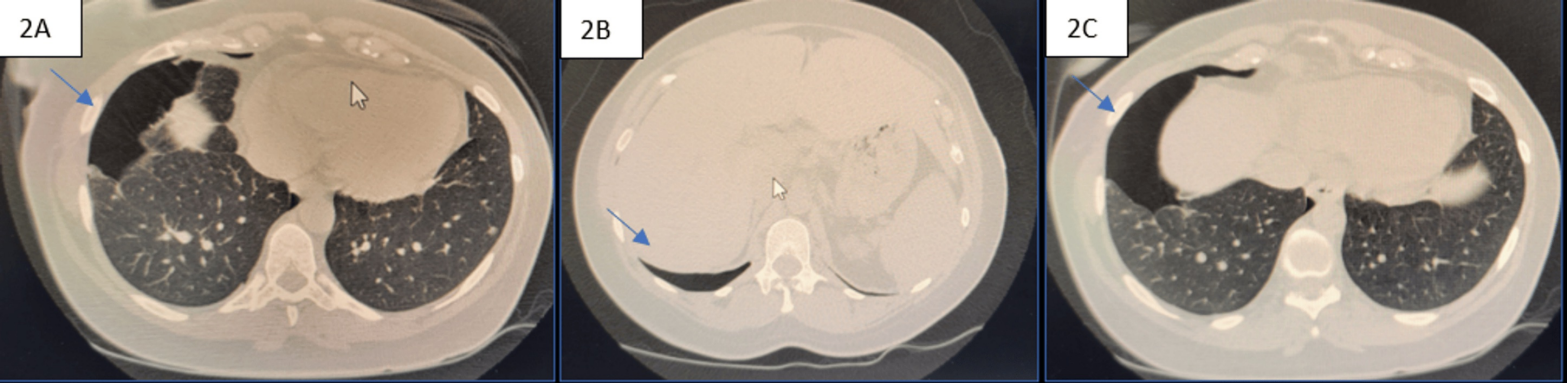
Non-contrast chest CT. 6/19/24 at 3:34 am: loculated right pneumothorax in the mid-lower lobe regions with a maximum thickness of 4 cm, associated with adjacent parenchymal collapse. A small loculated pocket of pneumothorax in the right apical region with underlying pneumo-atelectasis. Tiny subpleural parenchymal nodule in the left lower lobe.

Non-contrast CT scan of the chest showed a loculated right pneumothorax in the mid and lower lobe region with maximum thickness of 4 cm, associated with adjacent parenchymal collapse, a small loculated pocket of the pneumothorax in the right apical region with underlying pneumo-atelectasis, and a small subpleural parenchymal nodule in the left lower lobe (Figure 2). As a pneumomediastinum was not seen on non-contrast CT, it was most likely not present.

Labs showed an elevated alkaline phosphatase level at 184 U/L. Otherwise, no significant lab values were noted. The patient was initially placed on oxygen via a nasal cannula and then switched to a non-rebreather mask at 10 L when chest X-ray showed progression of the pneumothorax with suspected pneumomediastinum. The patient’s peripheral oxygen saturation remained above 94%, and she clinically improved. Serial chest X-ray also showed improvement of the pneumothorax, without continued progression; hence, the patient was switched back to oxygen via a nasal cannula before discharge. The patient remained stable, and peripheral oxygen saturation remained at 100% the entire time on oxygen therapy. No additional intervention was required as the patient clinically improved on her own. The total admission was for two days.

## Discussion

Thoracic endometriosis is an uncommon and rare disorder that can cause a variety of non-specific symptoms, including cough, shortness of breath, and pleuritic chest pain [2]. The most common clinical presentations include catamenial pneumothorax (occurring in about 73% of cases), catamenial hemothorax (occurring in about 14% of cases), catamenial hemoptysis (occurring in about 7% of cases), and pulmonary nodules (occurring in about 6% of cases) [6,7]. Catamenial pneumothorax is the most common presentation in as high as 65-80% of thoracic endometriosis syndrome cases [8].

Several hypotheses have been proposed regarding the etiology of thoracic endometriosis. The most commonly suggested etiologies suggest auto-transplantation of endometrial tissue either via retrograde menstruation or through the transdiaphragmatic passage of air from the genital tract or fallopian tubes [2,9,10]. Transdiaphragmatic passage of air may occur because of a defect permitting endometrial seeding via contractions of the uterus during menstruation or intercourse, in addition to the absence of a cervical mucus plug during menstruation. An autopsy review of cases with pleural and diaphragmatic implants noted an exclusive predilection of lesions to be on the right side. This supports the theory of transperitoneal-transdiaphragmatic migration of endometrial tissue due to the preferential flow of fluids from the pelvis to the right subdiaphragmatic area (i.e., through the right paracolic gutter, as congenital diaphragmatic defects exist predominantly on the right side of individuals) [10]. Both air and tissue have been hypothesized to travel through diaphragmatic defects, especially in cases with right-sided symptoms. Endometrial tissue present in the thoracic cavity or elsewhere responds to the endocrine hormones of the ovarian cycle, leading to bleeding simultaneously with menstruation.

The micro-embolization or metastasis theory can explain the lack of diaphragmatic lesions in cases that have a bilateral presentation. This theory precludes the need for implants to cross the diaphragm, traveling through either the blood or the lymphatic systems [5]. It is postulated that micro-embolization occurs after trauma or uterine procedures through the vascular systems, which is why they are bilateral [9,10]. Other theories suggest spontaneous rupture of blebs, causing pneumothoraces, or coelomic metaplasia, causing the differentiation of pluripotent cells into endometrial tissue [2,10]. An additional theory of alveolar rupture caused by prostaglandin-induced bronchiolar constriction or a check-valve mechanism exerted by bronchiolar endometrial implants has also been hypothesized. Specifically, prostaglandin F2-alpha has been proposed to cause vaso and bronchospasm, resulting in alveolar rupture and pneumothoraces [2,5,10].

Our patient had high alkaline phosphatase levels, possibly consistent with her recent pregnancy. In pregnancy, as the placenta releases alkaline phosphatase, a high value would be considered normal early in the postpartum period, perhaps suggesting why the patient had an increased value. There are no studies suggesting a correlation between high alkaline phosphatase levels and thoracic endometriosis. Cancer antigen 125 (CA-125), a tumor marker associated with epithelial ovarian cancer in postmenopausal women, has long been associated with endometriosis in young healthy women. An elevated CA-125 level in females with spontaneous pneumothorax should raise suspicion of thoracic endometriosis in the appropriate clinical context [9]. No other lab test has been determined to aid in the diagnosis of thoracic endometriosis. The diagnosis is made through consideration of the history of the patient, especially catamenial symptoms, and supported by imaging or direct visualization of the endometrial lesions during video-assisted thoracoscopic surgery (VATS) or medical thoracoscopy [9]. Differential diagnoses of thoracic endometriosis might include pulmonary embolism, pulmonary infections, other causes of pneumothorax, hemothorax and hemoptysis, and lung cancer. A high index of suspicion is first needed for the early diagnosis of this rare disease, which explains the later median age of thoracic versus pelvic endometriosis. Early diagnosis of thoracic endometriosis is important because it can lead to severe complications, such as recurrent pneumothorax and hemothorax, potentially causing respiratory distress, and, if left untreated, could lead to debilitating or life-threatening symptoms.

Diagnosing thoracic endometriosis may start with a chest X-ray or a non-contrast CT scan of the chest. Imaging may show findings of pneumomediastinum, ground-glass opacities, bronchial wall thickening, thin-walled cavities within the lung parenchyma, pleural or parenchymal nodules, pneumothoraces, pleural effusions, cavitations, or bullous formations [2,11]. Definitive diagnosis may be made with a high-resolution CT scan; however, VATS is the gold standard for diagnosing thoracic endometriosis. It is used both for diagnostic and treatment purposes. Video laparoscopy, a gynecologic approach, or VATS, a thoracic surgery approach, may be used for definitive diagnosis. Commonly reported intraoperative findings include diaphragmatic lesions in 38.8% of cases; endometriosis of visceral pleura in 29.6% of cases; discrete lesions such as bullae, blebs, or scarring in 23.1% of cases; and no findings seen on visual exam in 8.5% of cases [2]. Nodules on the surface of the diaphragm, if present, have been described as small and blue-gray [11]. The diagnostic results vary per case, and some cases have no visual findings intraoperatively. More than 60% of affected patients have been noted to require thoracotomy or thoracoscopy as part of a diagnostic approach [12]. Both diagnostic imaging and tissue sampling have been known to have inconsistent results, so biopsies are not required for diagnosis. Histological confirmation of endometrial tissue only occurs in 53-75% of suspected cases [11]. If initial imaging results are uncertain, additional imaging should be performed both during menses and mid-cycle to compare the findings [2].

Medical management is typically attempted as the first-line treatment. This includes the use of anti-gonadotropic agents to help decrease the production of endogenous estrogen. A hypoestrogenic state attempts to prevent proliferation of all endometrial tissue, including the ectopic tissue, by interfering with the hypothalamic-pituitary-ovarian axis [2,12]. Through suppression of this axis, menopausal-like symptoms may result, along with an increased risk of osteoporosis. Surgical management is used when medical management fails or is contraindicated. VATS has a 32-55% symptom recurrence rate in patients with thoracic endometriosis despite continuing hormonal therapy following surgery. Typically, even when surgery is considered, hormonal treatment is continued pre- and postoperatively for 6-12 months as the combined treatment approach appears to have a lower recurrence rate of symptoms [9]. Additional interventions such as pleurodesis, either chemically (talc or tetracycline) or mechanically (pleural abrasion or partial pleurectomy) have been shown to help decrease the recurrence rate of pneumothorax after VATS in 20-25% of cases when compared to cases that did not have pleurodesis at the time of surgery [2]. Depending on the extent of endometrial tissue or if perforations are present, a diaphragm resection, wedge resection, partial pleurectomy, lobectomy, open surgery, or hysterectomy with bilateral salpingo-oophorectomy, which is thought to prevent re-seeding of tissue from the pelvis, may be considered depending on where the ectopic endometrial implants are located and the degree of involvement [5,7,10-13].

Surgical, hormonal, and combination treatment approaches have been noted to have variable results in both short- and long-term outcomes [10]. Even with a combined approach of surgical and hormonal therapies, recurrence rates have been noted to be between 8% and 40% [13]. The rare diagnosis of thoracic endometriosis appears to show wide divergence in presentation, diagnostic imaging findings, and treatment techniques, as well as variability in recurrence rate and treatment methods among patients. Although there are theorized modalities for the pathophysiology of thoracic endometriosis, one theory does not fully explain the etiology of the disease in its entirety.

## Conclusions

This case describes a spontaneous pneumothorax from thoracic endometriosis in a postpartum female, a unique presentation not yet reported in the literature. The pathogenesis of thoracic endometriosis is not fully understood, emphasizing the importance of adding case reports to the literature. There are a variety of theories proposed, but none that fully explain the pathogenesis. This case presented two unique diagnostic challenges. First, thoracic endometriosis is defined as having catamenial exacerbations, and this case occurred postpartum in a relatively hypoestrogenic amenorrheic state. Second, the patient presented with pneumothorax on the right side despite the recent trauma of a uterine procedure. This suggests that the micro-embolization theory may not fully explain her disease. Further research will likely lead to a better understanding of the etiology of thoracic endometriosis cases.

## References

[1] Foti PV, Farina R, Palmucci S (2018). Endometriosis: clinical features, MR imaging findings and pathologic correlation. Insights Imaging.

[2] Nezhat C, Lindheim SR, Backhus L, Vu M, Vang N, Nezhat A, Nezhat C (2019). Thoracic endometriosis syndrome: a review of diagnosis and management. JSLS.

[3] Joseph J, Sahn SA (1996). Thoracic endometriosis syndrome: new observations from an analysis of 110 cases. Am J Med.

[4] Agossou M, Sanchez BG, Alauzen PH (2023). Thoracic endometriosis syndrome (TES) in Martinique, a French West Indies Island. J Clin Med.

[5] Nguyen K, Nudelman BG, Quiros J, Cortes M, Savu C (2023). Catamenial pneumothorax: a rare diagnosis among menstruating women. Cureus.

[6] Nam Y, Miodownik H, Ramanathan R, Viswanathan A, Dweck E (2023). A case of thoracic endometriosis presenting with tension catamenial hemothorax. Chest J.

[7] Santhyadka G, Chavko R, Ogirala R (2006). Thoracic endometriosis: a case report. Chest J.

[8] Macmillan A, Tan T, Jordan K (2018). Thoracic endometriosis presenting as recurrent right sided bloody effusion: a case report. Chest J.

[9] Velagapudi RK, Egan III JP (2021). Thoracic endometriosis: a clinical review and update of current and evolving diagnostic and therapeutic techniques. Curr Pulmonol Rep.

[10] Alifano M, Trisolini R, Cancellieri A, Regnard JF (2006). Thoracic endometriosis: current knowledge. Ann Thorac Surg.

[11] Hernandez FV, Purrman KC, Oliver AL (2021). Catamenial hemopneumothorax: an unusual presentation of spontaneous pneumothorax. ACS Case Rev Surg.

[12] Ziedalski TM, Sankaranarayanan V, Chitkara RK (2004). Thoracic endometriosis: a case report and literature review. J Thorac Cardiovasc Surg.

[13] Pham K, Penfold D, Kakkar A (2022). A complicated case of thoracic endometriosis with catamenial hemothorax and concomitant pulmonary embolism. Chest J.

